# “My life after stroke through a camera lens”- A photovoice study on participation in Sweden

**DOI:** 10.1371/journal.pone.0222099

**Published:** 2019-09-11

**Authors:** Karin Törnbom, Jörgen Lundälv, Annie Palstam, Katharina S. Sunnerhagen

**Affiliations:** 1 Research group for Rehabilitation Medicine, Section for Clinical Neuroscience, Institute of Neuroscience and Physiology, Sahlgrenska Academy, University of Gothenburg, Gothenburg, Sweden; 2 Centre for Person-Centred Care (GPCC), University of Gothenburg, Gothenburg, Sweden; 3 Department of Social Work, University of Gothenburg, Gothenburg, Sweden; USC Keck School of Medicine, Institute for Global Health, UNITED STATES

## Abstract

**Background:**

An increasing number of people with stroke live in their communities, yet the understanding of how their reintegration into society can best be facilitated is incomplete. If needs are not sufficiently met and difficulties overcome, it may result in limited participation and decreased life satisfaction for this group. We aimed to understand life after stroke through the lens of participants’ cameras, and hence their views and experiences guided this study.

**Methods:**

By the means of photovoice, an action research method, this study was conducted in a collaborative format with six women and five men after stroke. Participants photographed in everyday life for up to four weeks and then met to discuss all images in a focus group setting. Subsequently, participants gave feedback on the method and discussed the upcoming photography exhibition. All photos and the three focus group discussions were analyzed using a thematic analysis with an inductive approach.

**Results:**

In the focus group discussions, life after stroke were conceptualized through five main themes: a driving force to participate in society; managing everyday life through inventiveness and persistent training; insufficient healthcare and rehabilitation in the long-term perspective; finding meaningful relationships and activities in daily life. Participants’ voices are made clear through selected photos, which aim to present each theme and make results easier to understand.

**Conclusions:**

Participants found new ways to approach everyday life situations and had thereby regained a sense of control in life. However, it was evident that psychological processes towards adaptation were hindered by depression and that some individuals felt alone in an ongoing struggle. Additionally, available interventions a long time after stroke were not flexible enough to address all participants’ needs.

## Introduction

Stroke is one of the main causes of adult disability worldwide, with the greatest health consequences coming from a variety of physical, cognitive and emotional long-term impairments [1, 2]. Medically advanced acute care therapy [3] and modern rehabilitation after stroke [2] have increased the number of community dwelling persons with stroke [4], and in Sweden around 75% of stroke patients are discharged to their homes [5]. Stroke consequences are complex [6], and qualitative research has shown that in a long-term perspective, many individuals after stroke feel unsupported and abandoned [7, 8]. Nevertheless, participation and reintegration into society are the main objectives of many policy documents [9], and highly important both for the individual and for society [10, 11].

Photovoice is recommended as a highly participatory method that comes from the action research tradition, developed to empower participants by giving them a broader influence over research processes [12, 13]. To begin with, the main purpose with photovoice was to give voice to marginalized societal groups [14, 15], and in the last fifteen years it has been increasingly popular for visualizing the lives of people with physical disabilities [16].

An increasing number of people living with stroke, means higher demands for appropriate long-term rehabilitation services, and community reintegration programs that are evidence based [17]. As research participants are the sole authorities of knowledge about their life, qualitative research becomes grounded in subjects that are meaningful to those it concerns [4, 12, 18]. Photovoice gives participants the opportunity to photograph their identified subjects or environments, and to further discuss them in a group [4]. Choosing motifs that symbolizes what you really want others to know about life after stroke promotes reflection and an action oriented way of thinking about your life [13].

An additional gain with this method lies in how results are presented. By looking at photographs, the reader sees the world through the same lens as the participant and by reading the accompanying narratives they will understand more about each participant’s life [18]. Displaying results through photography is a pedagogical tool which makes them accessible for those outside academia. Results are easily grasped by policy makers and different professional groups, as well as for the general public, which can in turn initiate political discussions that may lead to improvements for people with stroke [13].

Although several studies have used photovoice in disability studies generally [16] very few have used it after stroke [4, 19–21]. To our knowledge, no photovoice study after stroke has been conducted with a Swedish study group before. Aside from being a highly participatory method that focuses on subjects chosen by participants this would be an important contribution to the research field because results are clearly demonstrated by visual presentation. This makes results available for people outside the research field, which may further promote the development and improvement of participation for this group.

The aim of this study was to explore the experience of everyday life after stroke and potential aspects of participation through the photovoice method.

## Methods

### Study design

This is an explorative, qualitative study with a photovoice design. The photovoice method is recommended to be used as a highly participatory method [15] and comes from the tradition of action research [22]. According to this, participants should be actively involved in the research processes, and the power of hierarchy should be genuinely respectful, open and democratic [23].

Photovoice is a method, where participants photographs and accompanying narratives are the foundation for data analysis [12]. The photovoice method promotes empowerment by encouraging participants to be engaged in active forms of reflection about their daily lives and circumstances [12, 13]. This study followed the procedure for conducting photovoice studies as outlined by Wang and Burris (1997) [15], and synthesized by Sutton-Brown (2014) [13]. The Consolidated criteria for reporting qualitative research (COREQ) guidelines were used [24].

In a focus group setting, participants had the opportunity to share their stories through photographs, and thus important aspects of their lives were communicated in a clear, visual format [4]. The focus group design allowed for participants to reflect with others about the broader meanings of selected photographs [12], and to find personal strength through these reflections [18]. Thereby, participants were provided with opportunities for growth in assessing and communicating important aspects of their lives [12].

Authors conducting this research consisted of: KT, a female doctoral student with a Masters in social sciences and previous experience and knowledge within the field of qualitative research. The second author JL is a male associate professor in social work, with extensive experience and knowledge in the field of qualitative research. The third author, AP, a female with a PhD in medicine and a registered physiotherapist with previous experience in performing qualitative studies. The last author (KSS) is a female MD, stroke specialist and professor in rehabilitation medicine, with considerable experience in stroke rehabilitation.

### Sampling and participants

A purposive recruitment procedure was undertaken in order to obtain a heterogeneous study group, and thereby attain a rich variation in results. Individuals were chosen with respect to gender, age, ethnicity, level of education and stroke type [25]. Inclusion criteria were; having had a first time event of an ischemic or haemorrhagic stroke, residing in the Gothenburg urban area and being ≥18 years of age.

Firstly, individuals with stroke, who had previous contact with researchers at the department were contacted through a selective procedure. Of 14 persons contacted, seven individuals (one person with aphasia) agreed to participate. Then, the local stroke association and various other organizations who arrange rehabilitation for persons after stroke were visited and informed about the present photovoice study. Two persons were recruited from these organizations. Information about the study was posted on the wall of two Facebook groups with members with acquired brain injuries (stroke or other) in Sweden. Two members of these groups were interested and contacted the first author (KT) by phone to notify their interest in participating. Eleven individuals, with differences in age, ethnicity, level of education, type of stroke agreed to participate, hence the variation in characteristics we aimed for had been achieved and so the study began.

### Data collection

Participants were invited to a preliminary meeting about the photovoice method, ethics of photography in public and camera training. Three participants declined to attend the meeting and were visited by the first author KT to receive the same information at home. All participants provided written informed consent at this preliminary meeting. Participants were instructed to fill in a form about demographics, which is presented in Table 1, together with information about stroke type and severity that were retrieved from medical charts. None of the participants reported visuo-spatial neglect, visual impairments or field cuts that influenced the photography. However, this was not clinically tested.

**Table 1:** Characteristics and clinical assessment of study participants, n (11).

***Age***	
Mean years, SD (min-max)	58, 11 (34–70)
***Gender***	
Women/Men	6/5
***Born in Sweden***	
Yes	9
No	2
***Living situation***	
Alone	6
Cohabiting	2
Married	3
***Mobility aid*** (more than one is possible)	
No mobility aid	6
Walking stick	3
Walker-rollator	2
Motorized wheelchair (outdoors)	2
***Ability to drive a car after stroke***	
Yes	3
No	8
***Stroke subtype***	
Ischemic stroke	5
Intracerebral hemorrhage	6
***Lesion side***	
Left	5
Right	5
Bilateral	1
***Stroke severity* (NIHSS**^**&**^ **at admission)**	
Mild (0–4)	6
Moderate (5–15)	1
Severe (16–42)	4
***Years since stroke***	
2–4	3
5–7	3
9–12	3
>14	2
***Aphasia***	
No	10
Yes	1
***Vocational status***	
Retired	2
Sickness/activity compensation*	4
Sick-leave^¶^	1
Working full-time	4
***Level of education* (years in school)**	
Primary school (9)	1
High school (12)	4
High school and training courses within a profession (12–14)	3
University (>15)	3
***Children***	
Yes	10
No	1
***Occupations***	Self-employed mechanic Social worker (sick-leave) Software developer Creative designer Health care administrator
***Occupations prior to retirement or sickness compensation***	Lawyer Pediatric neurologist Performer in creative arts Project manager Business secretary Psychiatric nurse

*Sickness compensation is commonly known by its previous term “early retirement”, it is granted if no improvement in working capacity is to be expected ever again.

¶ Sick-leave is a temporary form that is financed through sickness benefits

& NIHSS, National Institutes of Health Stroke Scale

Through the relatively free frameworks in interpreting research aims, the photovoice method offers participants an active position which enables them to show their own agenda through narratives and photographs [13]. Participants were asked to reflect about their daily lives, and to take pictures of situations, persons, or anything else which they considered meaningful. They were invited to share both negative and positive aspects of their lives after stroke. Participants were encouraged to be personal, and to think freely when choosing motifs for their photography [15, 18]. They were not told to take pictures of their participation, even if this was something that we aimed to study. The concept of participation might be considered vague and difficult to understand, and as we wanted to prevent confusion and unnecessary interference from the research team, this concept was avoided in the instructions. An overall objective was to make participants feel as free as possible when choosing their photography motifs [18].

All participants were given a disposable camera. However, due to the limited quality of photos taken with this type of camera, they were encouraged to use their mobile phones or a camera of their own. Three participants chose to use the disposable camera and eight used their mobile phones. Participants were asked how much time they needed for taking photos, within a study frame of 1–4 weeks. Most participants had taken all their photos within two weeks, although two participants completed their photography in four weeks, which they considered enough time. They were instructed to take as many, or few, photos as they wished, and then select photos for their focus group presentation. They were also instructed to produce a short written description of each photo [13]. All photos were developed in a photo store on photo paper (matte), and were handed out to each participant by the first author ahead of the focus groups.

#### Focus groups

Three focus group interviews were conducted by the first author (KT) as the moderator. Interviews were performed during November to December 2018 and lasted for an average of about 80 minutes. Interviews were transcribed verbatim by KT, close to the date of the interview so that initial reflections and thoughts about discussions were fresh.

Using self-selected photos in interview situations allows participants to produce narratives, focused on issues central to them [15]. The focus groups provided participants with space for self-reflection, and to create meaning together with other group members [12]. In photovoice, the focus lies on participants’ personal interpretation of photographs [15]. This is important when considering the optimal design for sharing and presenting photographs. In the present study there were a low number of focus group members; three in two groups and five in one group. Small groups were chosen, so that all participants could be actively involved in discussions. It was also considered important to discuss all the photos, and to achieve this before anyone got too tired or lost their ability to concentrate.

An additional intention of the smaller groups was to make participants feel comfortable sharing their personal narratives. It turned out that group members contributed greatly to create an atmosphere that was comfortable for sharing. The focus groups were characterized by compassion, where members showed interest in each other’s concerns, gave practical advice and used humor to relieve tension. These supportive interactions promoted discussions, and we believe that all participants understood that their individual contribution were really important for the study results.

A structured and detailed way to present photos is commonly used in photovoice studies [13]. This model for presenting photos was introduced in the focus groups. However, participants agreed that they wanted to discuss their photos more freely, and so they did.

To begin with, participants were asked to present themselves to the other group members. They were then asked to select up to ten photos for discussion, together with their written reflections about each photograph. All photos were arranged by participants in front of them [13]. One participant would begin to describe a photo, and the other members joined in by sharing their interpretations and thoughts within the same topic. Through this procedure, photos about similar topics were debated simultaneously, and participants did not have to switch focus from one topic to another. Participants were involved in the process of organizing photos for certain themes, which helped to democratize the research process [13].

#### Ethical considerations

The study followed the Helsinki declaration and was approved by the Regional Ethics Committee in Gothenburg (EPN) 20181011, Dnr: 800–18. Participants gave written informed consent prior to the focus-group interviews. It was clearly communicated that photos in this study were going to be presented and published in a research article, and that participants had to notify us if they did not want their photos to be made publicly available. The individuals in this manuscript has given written informed consent (as outlined in PLOS consent form) to publish these case details. All PLOS consent forms have been securely filed in the participant’s case notes. Prior to signing the informed consent, participants were informed that no financial compensation would be given in this project.

### Data analysis

The six phases of thematic analysis as described by Braun and Clark (2006) [26] (see Table 2) have been followed in the analysis. An inductive approach with an essentialist/realist epistemology was chosen to enable an open, yet straightforward way to theorize meaning in the analysis. Additionally, the poly textual thematic analysis for visual data was used as inspiration for how to approach an analysis that includes images [27]. In this context, our aim was to grasp how images were interpreted by participants, rather than what they meant to us as researchers. Hence, photos are analyzed through the participants’ narratives, and extensive interpretations are in the eye of the beholder. The transcribed focus group interviews were the basis for analysis. Photos were mainly used to emphasize and visualize what participants wanted to say–in making their voices clear. Images were intentionally used as tools to unfold the broader meanings of themes and make them easier to grasp.

**Table 2:** An overall guide of the thematic analysis as outlined by Braun and Clarke [26] including features of an image-analysis [27].

Phase 1	Familiarize yourself with data. Read and re-read transcripts and look at the images many times (singly and in different groupings) to generate an initial list of ideas about what is in the data [27]. Images are analysed by accompanying short descriptions, made by participants.
Phase 2	The production of initial codes from data. Data was coded to identify particular features of the material. These features with accompanying photos are identified as potential codes [26, 27].
Phase 3	Analyse codes to see how these can be combined to form overaching themes and subthemes. Take care to study the feature of the images, and how these might visualize one or more themes [26, 27].
Phase 4	Reviewing themes. A set of candidate themes are devised and this stage involves refinement of themes. Which extracts do not fit at all? Are all themes distinct? You might need to rework the themes. Generate a thematic map of the analysis [26].
Phase 5	Defining and naming themes. Identify the essence of what each theme is about and determine what aspect of the data that each theme captures. Collated data extracts should be organized into a coherent and internally consistent account, with accompanying quotes. If there is any lack of clarity, redefine the themes that are identified. The object is to maximise differentiation in order to find distinctive features of the text and accompanying images. Identify what is interesting about the themes and why when labelling them [26].
Phase 6	Producing the report: It is important that the analysis provides a concise, coherent, logical, non-repetitive, and interesting account of the story the data tell within and across themes. Extracts need to be embedded within an analytic narrative that compelling illustrates the story that you are telling about your data, and your analytic narrative needs to go beyond description of the data, and make an argument in relation to your research [26].

In some photovoice studies [16], including the model that we used [13] participants were involved in larger parts of the analysis, for example, in producing codes and categories. This approach was not considered feasible in the current study. A qualitative analysis is time-consuming and requires a lot of work, and as participants did not get any financial compensation, it was considered unreasonable to ask for such a high level of engagement. However, the analysis was shared with participants for validation purposes, and participants were invited to suggest any changes [28].

To obtain a sense of the three transcriptions and to search for patterns of meaning in data, interviews were read and re-read several times by two of the authors separately (KT and AP). It is inadequate to interpret a quote alone, and therefore the material must be treated as a whole [27].

Authors (KT and AP) met to discuss and compare each other’s initial marked ideas for coding and began the more formal coding work. As the analysis was data-driven, texts were approached with an open mind and many codes were located. Therefore, an essential part of the analysis was to consider how codes could be combined in forming overarching themes. Photos were helpful at this stage, symbolizing different potential themes. Themes and sub-themes were refined, collated and re-structured by two of the authors (KT and AP) until consensus was reached. The themes were then reviewed by all authors, with the aim to enhance the credibility of the analysis (KT, AP, JL and KSS).

Finally, an adequate thematic map was produced and the essence of each theme was determined and described. The findings were subsequently shared with participants for validation purposes, and they were invited to discuss and to give further interpretations of results. Participants were also contacted to give feedback about the study design, which is presented in the discussion. Finally, the dual voices of visual and spoken language were used in the results to reinforce participants’ narratives.

### Photo-exhibition

A part of the photovoice method is sharing photos and corresponding narratives with a public audience [12]. Participants were invited to discuss who their target audience should be, and to decide upon the format and design of an upcoming photo-exhibition. Participants emphasized that stroke is a public disease and at first hand they wanted common people to be the target audience. They said that “everyone has some type of connection with stroke and can benefit from knowing more about what everyday life after stroke is like.”

Participants also wished to reach out to local politicians and policy makers with enough power to make and enact policy surrounding infrastructure and accessibility for persons with disabilities. The idea was to choose photos associated with accessibility, and to make a special exhibition of these at two municipality halls. The municipality halls, as well as local museums, school and librarian administrators were contacted about an upcoming photo exhibition (autumn 2019). At the time of writing this article (spring 2019), nothing concrete has yet been decided.

## Results

Participants described the years following stroke as a process of regaining functions and resuming everyday activities to their liking. Adaptations towards a somewhat new way of living had taken place in the participants’ homes or communities. Their reintegration practices were influenced by physical inaccessibility, somewhat rigid social systems and a general public lacking in understanding. Nevertheless, physical and mental adaptations, together with rehabilitation exercises, had resulted in increased participation for almost everyone.

Photos are presented under each theme, and additional photos can be found in the S1 Example images file.

### A driving force to participate in society

When being out and about in their community, participants were afraid of not being treated with respect and of falling or being hurt in traffic. Concentrating on walking at a steady pace on uneven ground, and at the same time keeping focus on traffic was physically and mentally demanding. Several emphasized that a goal for them was to walk more freely in their community. The feeling of freedom and joy from walking on even ground was also expressed;

*There’s concentration on what you are doing and your body parts… lots of traffic and there was no traffic light there*, *but traffic is coming from all directions*. *And you think*, *oh*! *There was a hole there*, *and you have to concentrate on the ground*, *even though you shouldn’t really be looking down… but in one part of town (shows the photo) it was fantastic*. *You could walk without holes*. *I felt free and really happy* (Woman 63 years).

**Fig 1:**
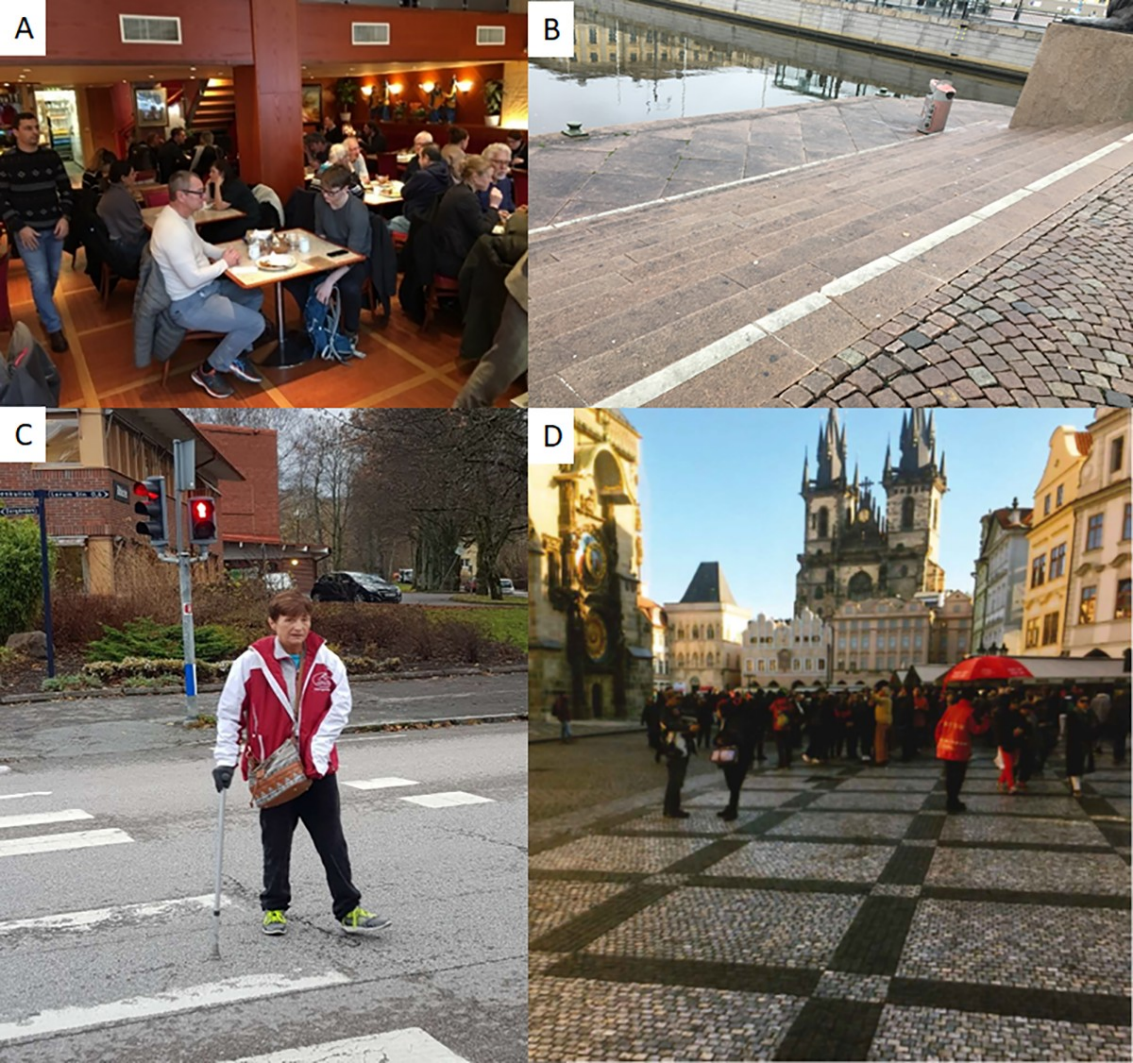
A. Restaurant:”Nice but too loud!” B. Stairs by the water: “Stairs with no handrails are scary” C. Crossing the road: “I cannot pass before the light switches to red” D. Town with church: “A desire to walk every day on a flat surface like this”.

It was generally stated that, people with disabilities make a lot of effort just to get out of their houses to actively participate in society, see Fig 1. In addition, participants were concerned that people in general did not seem to take any notice of how much effort they exerted on a daily basis. Disabilities that were invisible in the eye of the general public, for example; insufficient balance, poor vision or hearing, and difficulties concentrating in social situations were said to reinforce this feeling of being met by prejudice or not treated with respect by people in general;

*People expect that a relatively young person shouldn’t have issues with moving*. *When you were in large groups of people that was really uncomfortable*. *It is really unsafe*. *Then*, *it was good to be able to sit in the wheelchair* (Woman 55 years).

*It can be frustrating when you are out… and there’s no one who notices*! *That you’re mentally exhausted or have memory problems*. *It’s actually really important that people are made aware of this… I can’t walk to the bus by myself*, *there are roots that stick up*. *Many times it’s actually been really difficult*, *and people around you don’t get it*! (Woman 63 years)

In social situations some participants had to concentrate hard when several conversations happened simultaneously. With too much sensory input it became impossible for them to follow a conversation. Difficulties concentrating and communicating with several people at once had resulted in them avoiding going to restaurant visits or similar. Social interactions, which included many people or went on for several hours, made participants concerned about getting worn out, and having to rest for a long time afterwards;

*I have about two hours* … *or rather my capacity of good ability (of socialising) is two hours*. *But I am an expert at toughing it out*, *so I manage much more*, *but then I am just done*. *She (daughter) left on the Sunday and it was around the following Thursday that I started to come out of it (recover)* (Woman 57 years).

Nevertheless, the desire to come out and try to socialize often seemed stronger than their combined concerns. Participating in society was one way in which participants could obtain a sense of belonging and social identity. Participants used their own initiative to engage in activities, but also because they needed to get to work.

At time of the interview, four out of eleven participants were working (fulltime). Social interactions at work allowed participants to forget a little about stroke related impairments and the social aspects of work were described as rewarding. Working was also considered to be a meaningful part of life;

*I feel that if I hadn’t done it (my job) and come back*, *I wouldn’t have been sat here and been ‘okay’ like I am now* (Woman 57 years).

Two participants felt that they were not really managing working fulltime, but that their application for part time sick leave had been denied by the Swedish Social Insurance Agency. Continuously stretching beyond their capacity in order to meet demands that were set too high was described as “physically draining” and “exhausting”.

Strategies to get to work on time were to set the table with breakfast in the evening before and choosing clothes and laying out belongings (such as wallet/keys/phone) in advance. Taking breaks, resting at lunch or wearing earmuffs were strategies used to cope with fatigue during a workday.

### Managing everyday life through inventiveness and persistent training

**Fig 2:**
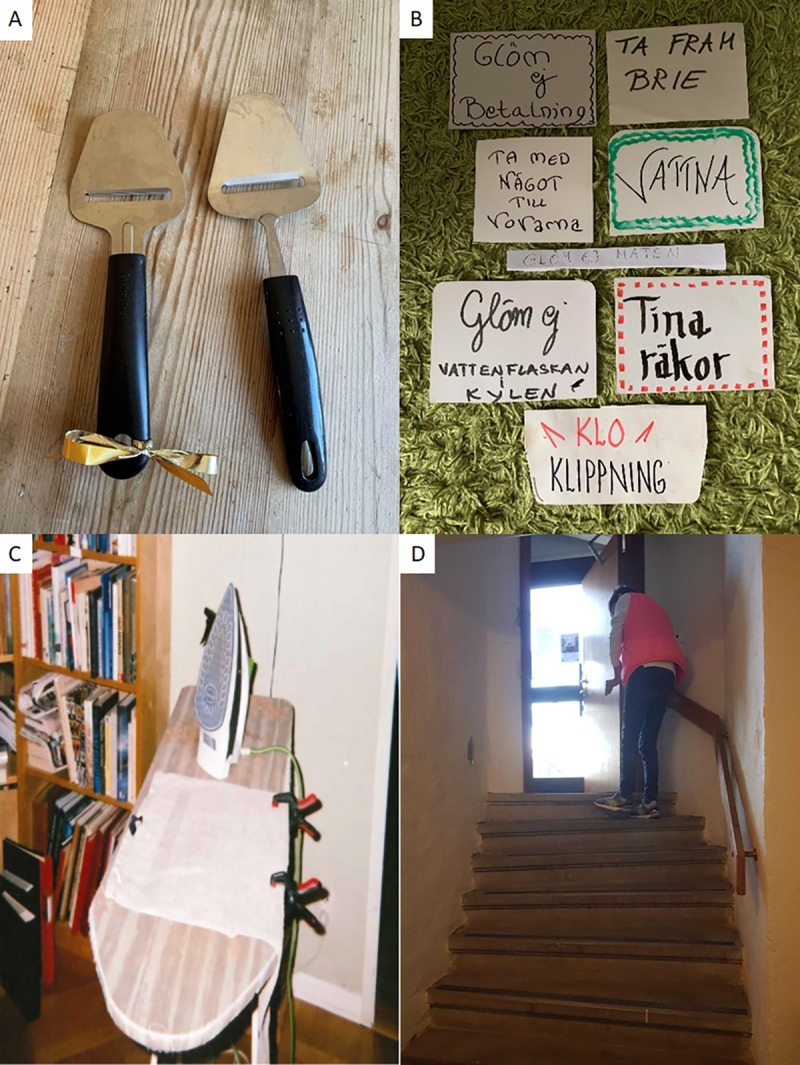
A. Perception: “A string on the cheese slicer. After stroke, I cannot tell them apart” B. To-do notes:”I put them in a shoe or on the doormat, so I don´t miss anything” C. Ironing board:”I fasten the fabric with one-hand clamps. My surrogate hands” D. Walking down stairs backwards: “If the handrail is on my affected side, I walk backwards”.

Participants had a lot of creativity when it came to problem solving, see Fig 2. Their approach consisted of never giving up and being inventive and creative in everyday situations. Writing post-it notes and put them in your shoes, walking down stairs backwards or using a one-hand operated clamp when ironing or cooking were a few examples of the inventive approaches;

*Often there are little tricks to solve a problem*, *you just can’t give up too soon…you have to be brilliant in difficult situations* (Woman 55 years).

*My cellar stairs have eleven steps and at the bottom is a radiator*, *and I can’t go downstairs if I can’t hold onto a railing*. *So that’s why I throw down the dirty washing and walk around (outside to an external entrance)*. *I go in through the cellar door entrance and then I can do my washing* (Man 70 years).

Consequences after stroke were also handled strategically and in a preventive manner. For example, not taking on more than one assignment per day to avoid fatigue or asking your next of kin when your memory failed. Through trial and error many everyday problems had been solved in a way with which participants were rather content.

Participants stressed the importance of taking their rehabilitation training seriously. Several trained physically daily, mostly balance, but also strength, fine motor skills and coordination. Even participants who had their stroke more than ten years ago still experienced some improvements through training. There were also examples of physical functions that had been regained through persistent training;

*Balance was something that was really hard at the beginning*. *I had really bad balance*. *But I worked out like hell*! *Oh how I have worked on my balance*! *To be able to go up that*, *and go up and down stairs and that sort of stuff* …*I can walk up and down stairs now without a problem*. *But it was hard work* (Man 48 years).

In general, insecurities and fears had decreased with improved balance, strength and concentration. Social interactions were now easier to handle thanks to improved speech and an increased ability to shut out surrounding noises. These small steps towards enhanced participation and a life with greater possibilities were referred to as “fantastic”, “wonderful” and “great”.

*The steps you take towards improvement… that is fantastic*! (Man 48 years).

### Insufficient healthcare and rehabilitation in the long-term perspective

Participants expressed a general disappointment with the healthcare system, its accessibility and follow-ups. Being allowed rehabilitation at a clinic six months or longer after stroke was said to be difficult. In order to receive help for stroke related problems, participants stated that you needed to come in earlier than six months after stroke, and that it was difficult to receive continuous and adequate help at all after this time point;

*But Sahlgrenska* (one of the largest University Hospitals in Sweden) *is not good and offering help*. *You get help for six months after the stroke*, *and then you get no more help*. *The speech therapy stuff I have had to sort out myself*. *Same thing with physiotherapy and other exercise* (Man, 67 years)

Participants preferred to do their rehabilitation training together with a professional who could acknowledge and encourage their efforts. In addition, they expressed uncertainties about how to manage stroke rehabilitation on their own, and what services you needed to be self-funded. Participants found it difficult to find the right information and make use of it. Although they had received a lot of information about post-stroke rehabilitation in the acute phase, they were not able to remember it several years later.

It was discussed that some parts of the healthcare system available for persons with disabilities were poorly designed to meet persons with special needs. Remembering appointments or finding locations were really complicated for some participants. Another difficulty for participants with fatigue or recurrent headaches was knowing a long time in advance (24 h) whether they could show up at a scheduled time or not. Cancelling too late repeatedly had resulted in bills for the visit, which had made participants avoid making further appointments.

*I looked up a stroke team and they were angry with me because I didn’t come to a booked appointment with a psychologist there*, *but I was asking for help with my memory…so at the last meeting they were saying “but you didn’t come here (for the appointment)”…and I have already talked about the fact that I have problems with my brain (remembering and having energy for stuff) but it wasn’t according to their policy*, *that you could fail to turn up* (Woman 53 years)

**Fig 3:**
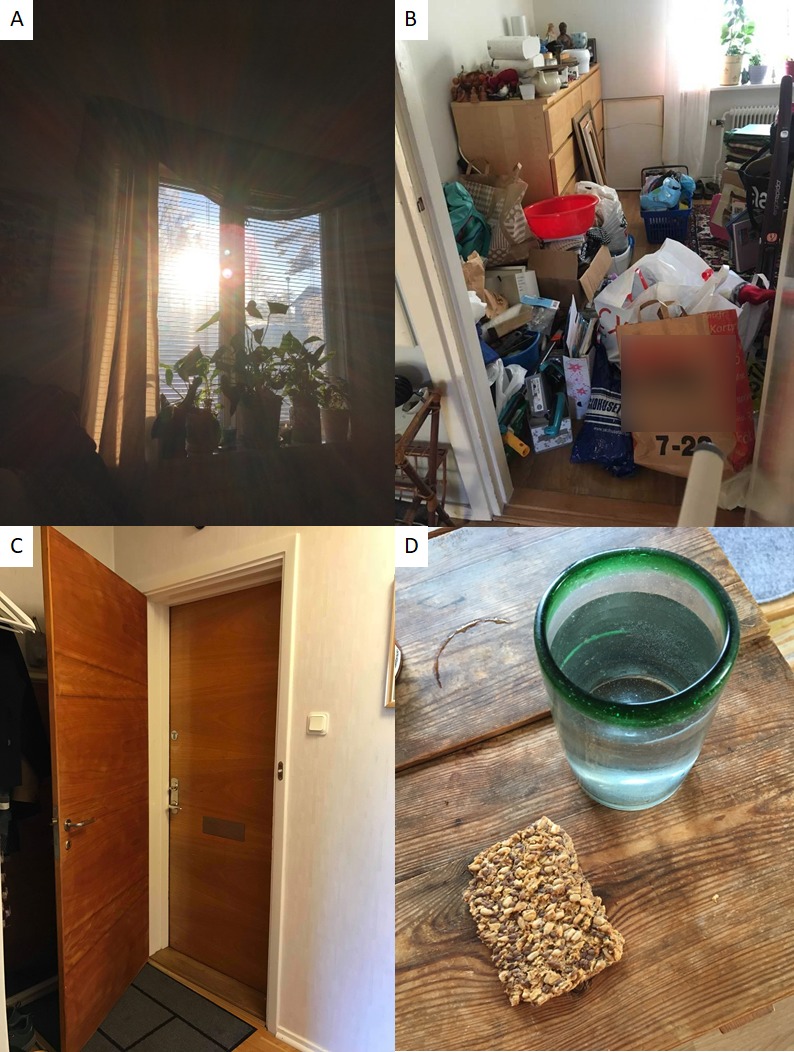
A. Window blinds:”This is what I see everyday. I am too tired to get out of bed” B. Hoardings: “I cannot manage to sort out my belongings” C. Door: “My front door symbolizes a huge obstacle for me to get out” D. Water and bread: “When my headache is severe I cannot cook proper food”.

When it came to some consequences of stroke, participants had adopted a resigned mood, see Fig 3. It was emphasized that headache post-stroke, fatigue or a poor primary internal or short-term memory were very hard to handle. Participants could not solve these problems by themselves and had therefore, in some cases and out of despair, sought help from companies of questionable quality offering solutions. For example, a bank loan was taken to finance a spiritual health course, which was afterwards met with great disappointment and increased financial difficulties. Such impairments, that participants had not accepted as parts of their lives made them depressed and anxious.

### Finding meaningful activities in daily life

It was stressed that taking part in activities of one´s choice made everyday life more meaningful;

*It was when I was at a handicraft course*, *then I could sit there for a whole day and not be tired afterwards*, *then I was just full of energy*, *because there was some kind of super focused space I got into*, *and it was like*, *really quiet all around me… in a really nice way* (Woman 33 years)

However, several were concerned about the difficulty in finding activities and stressed that they would have benefited from some (professional) help in doing this;

*And I didn’t know that it existed* (silversmithing) *when I had the stroke*, *and it was something that no-one told you about*. *But then I met her* (a friend) *in town*, *and she said “But why don’t you come to our rehabilitation group*?*…you can go there if you’ve had a stroke” And information isn’t given personally*, *so I didn’t know anything about it*. *But then she said “We have silversmithing*, *and I know that you would love it*! (Woman 65 years)

**Fig 4:**
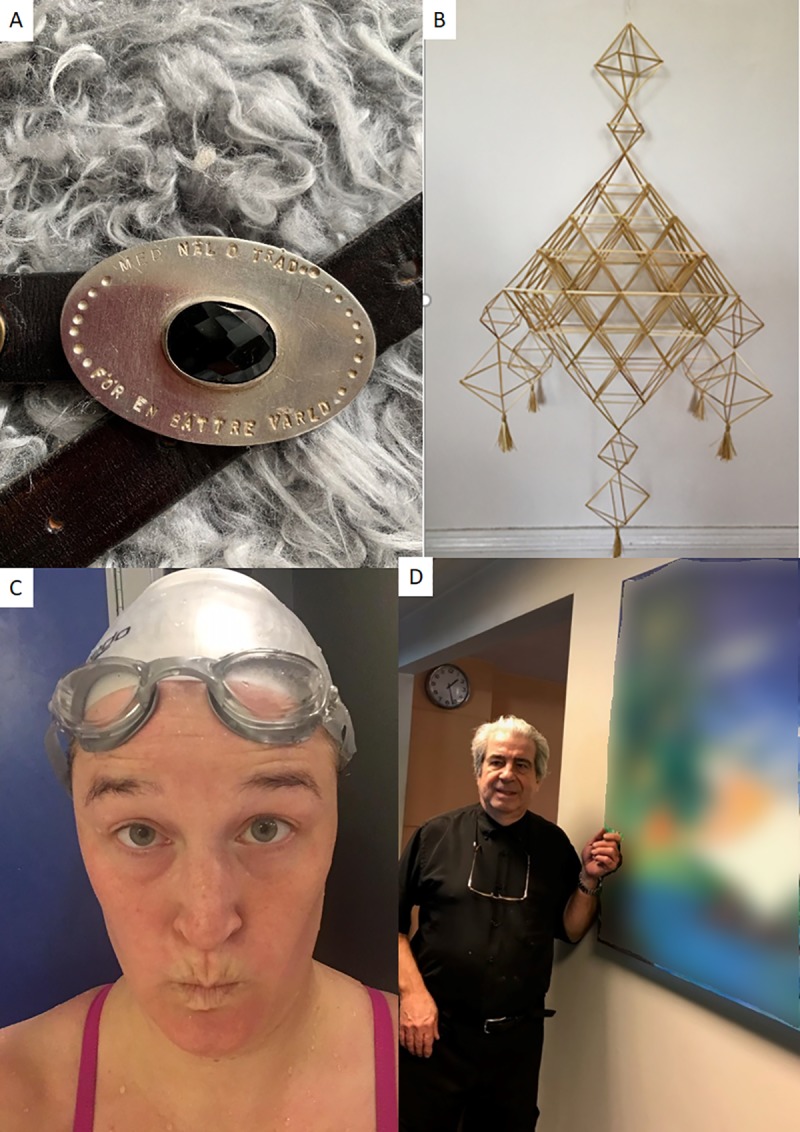
A. Silver-crafts:”A fascinating interest after stroke is making jewellery” B. Art with straws: “Making geometric art makes me totally focused and relaxed” C. Swimming: “When swimming you are not afraid of falling or losing balance” D. Art: “I very much enjoy looking at paintings after stroke. I get happy every time I see this”.

As mentioned earlier, participation was enhanced through diligent rehabilitation training that had improved physical and cognitive functions. Participants were keen to speak about the feeling of joy and relief that they had felt when body functions or activities were regained, see Fig 4.

*I thank God that it* (my hand) *has come back*. *I picked up a glass*, *and said “I’m going to drink”* (shows how she drinks). *I had to put the glass back down again*. *And I tried the whole time*, *and talked to my hand*. *“Come on*, *come on” and in the end I saw two fingers move*. *On the Friday I had a ball in my hand and I could throw it away… So I screamed* (of joy), *and the physiotherapist screamed*, *because it was so fantastic*! (Woman, 63 years)

Mastering pre-stroke activities helped strengthening participants’ self-esteem, and made them feel freer and happier in everyday life;

*To begin with*, *it was really unpleasant to hear all the noises in town*, *but I have practiced with screening it out… that there’s my daughter and me and we can look at shop windows and it works really well*. *I’ve worked on this*. *I have to get all the noise lower*. *And it took over a year until I started to get a handle on it*, *but it was really nice* (when I did) (Man 48 years)

Several participants were involved in new activities that had been initiated “thanks to the stroke”. This was partly forms of exercise to sustain body functions; like yoga, swimming or swimming freestyle. Exercising in water was mentioned as relaxing when struggling with poor balance, as you cannot fall. But also quiet recreations in forms of writing books, silversmith’s work, handicraft and art. Participants had been motivated by people in their surroundings to find new activities, and they had also wanted to participate in meaningful activities. Another aspect was the reduced fee when applying to a course for people with disabilities;

*I thought about all the benefits you can get* (from having had a stroke)*… I have the advantage that I have a small piece of silver jewellery here* (shows a photo), *that I have done myself at silversmithing*. *And I get it for a lower price*, *because it is normally quite expensive to learn silversmithing…But this was just fantastic*! *And I feel like I have got so much from the others who go there too* (Woman 65 years)

Several participants had started to write after stroke, either in forms of a diary or novels and even books. The writing was described as healing and a way of training cognitive abilities.

### Meaningful relationships in daily life

**Fig 5:**
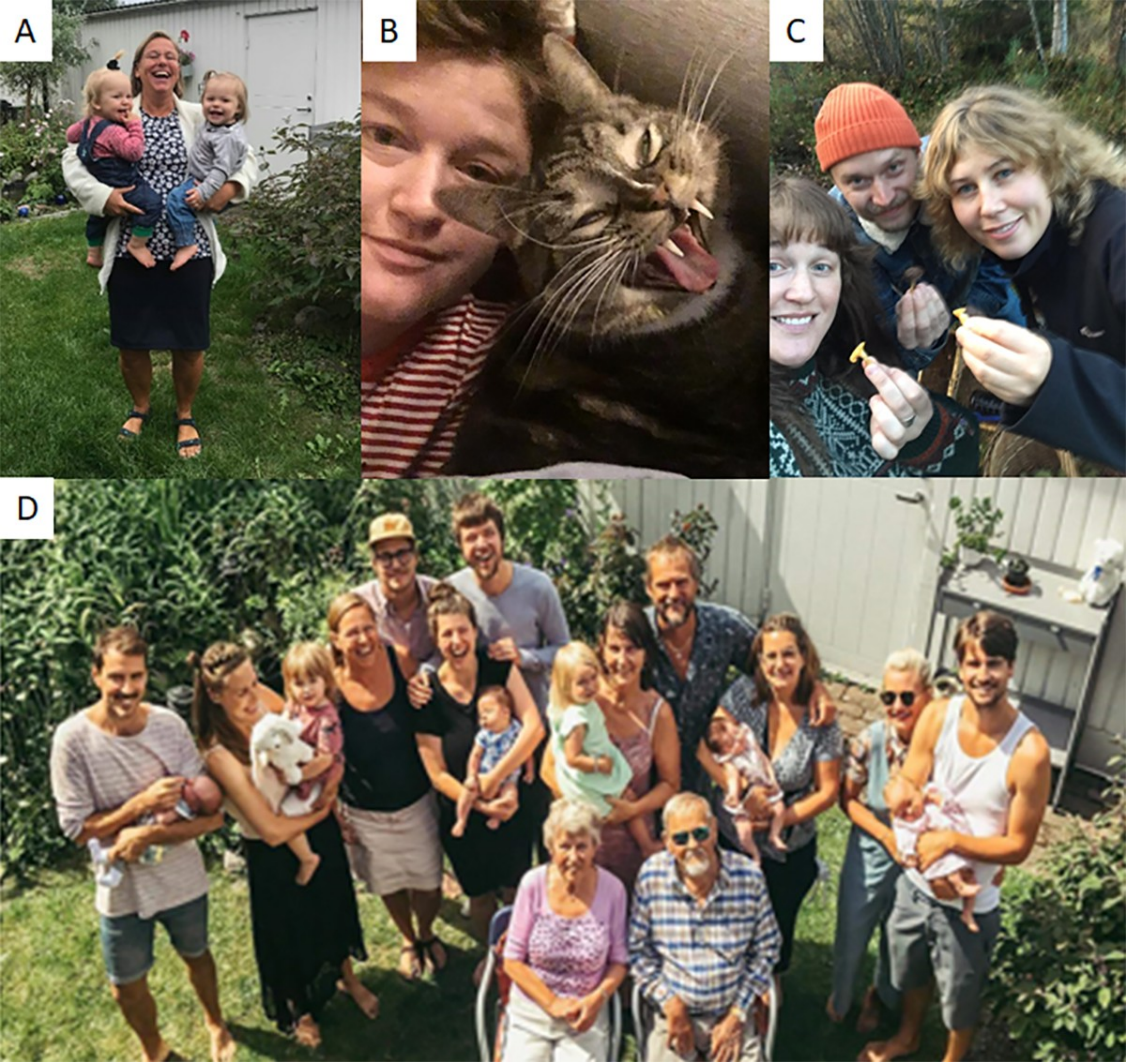
A. With grandchildren: “They are such joy!” B. Lady with cat: “Cuddling with my cat is like receiving unconditional love” C. In the forest with friends: “Being outdoors with friends is lovely” D. Picture of relatives: “My family–rewarding and challenging”.

Participants spoke mostly about their own responsibility in ensuring that daily life was spent according to their wishes. However, they had different strategies to promote mental energy so that hurdles in everyday life could be approached more easily, see Fig 5. Close friends, relatives and interacting with work colleagues were described as very meaningful parts of life. A majority of participants lived alone and relationships with pets were described as unconditional, relaxing and healing;

*To cuddle with pets is an excellent way to recover* … *cuddling with my cat has been lifesaving to me! And also, that pets don´t speak! So nice (Woman 33 years)*

*I feel like I am really calm because of the dog, when you pat and cuddle with them. And then you get the oxytocin. Touch gives you that good hormone*. *(Woman 63 years)*

Some participants received too little social and emotional support from family and friends. As a result, they felt lonely and abandoned in their struggle towards a better life. This loneliness was described as an ongoing grief and loss;

*You end up completely alone* (after having a stroke). *They disappear*! *A whole family can disappear*. *And that is a huge huge loss* (Woman, 52 years)

Participants agreed that hippotherapy had been important for their recovery. Aside from the warmth and the emotional connection with the horse, it was stated that strength and balance were trained efficiently through horseback riding. Riding a horse was also described as emotionally rewarding and empowering;

*I felt like a cowboy… and the heat coming from the horse makes you relax*, *it was an amazing experience for me*. *It also helped my physical abilities to come back*. *And in the end*, *I could ride the horse like I did before having a stroke* (Woman 57 years)

## Discussion

Photovoices after stroke were captured and revealed concerns about participation in everyday life. These voices were mainly about participants’ individual journeys towards adaptation and recovery. Persistent rehabilitation training, a strong will to solve problems in everyday life and not giving up had been important factors during this process. The concept of re-focusing, adapting old skills and changing the environment accordingly was found in a previous qualitative study [29]. Participants in our study had mostly reintegrated desired activities in daily life and accepted how life had changed to fit their “new” selves. For some this reconciliation between past and present selves was described as a transformation of a new identity that included changed abilities [29, 30].

Our findings illustrated difficulties in accessing the community due to physical and societal barriers. Both participants with visible and invisible disabilities felt anxious of not being met with respect and understanding from the general public. Mainly the city centers were said to be poorly designed with respect to accessibility, for examples cobblestones, uneven ground and tram tracks. That infrastructure is sometimes inaccessible for people with stroke disabilities has been shown before [4, 31]. Interestingly, participants felt motivated to go out, despite all of the associated anxieties. The desire to actively participate in one’s community was profound, but then again, required maximum efforts of concentration as well as physical and mental energy. It is probable that opportunities for participation could be facilitated through a more accessible community, with infrastructure designed with input from persons with disabilities [4]. In contrast, when voices of persons with disabilities are not considered when creating public spaces, it signals to them that their participation is less important and might enhance their feelings of being discriminated against.

Photovoices of this study displayed practical inventions in everyday situations (i.e. letting a one hand operated clamp work as a hand), that can be seen as solutions to adapt to stroke consequences through practice. The desire to participate in everyday life according to one´s wishes seemed to have been a motivating force towards recovery. Consistent with this finding, the desire to reintegrate with aspects of the identity that participants wanted to restore, was a guiding factor through the rehabilitation process [32].

Some participants had found new activities that they really appreciated. These new skills and interests were described with pride and joy, and it was obvious that participants did not considered them as compromises for living with stroke consequences. According to a study on self-management after stroke [33]; *individual capacity* as well as *social and environmental support* that strengthened the individual, were key components for such successful recovery. Our findings supports that narratives about mastering new activities or competencies also contain close and supportive relationships, and other various resources of importance on an individual level.

Participants who struggled with more severe, or a mix of, consequences on a daily basis emphasized that they had not yet accepted how their lives had changed after stroke. Consequences made them depressed and had led to resignation and social isolation. Participants did not know how to handle their unbearable symptoms and felt an ongoing distress and frustration over not receiving appropriate care. This state of mind can be related to a study that resonated about individuals who put their locus of control over health issues on “external others”, whom they saw as powerful (for example doctors, or special health care methods) [34]. It also indicates a certain vulnerability and hopelessness, which is in contrast to thinking that your own actions are the most important factor in determining your course of health [34].

Participants who expressed dissatisfaction with both themselves and their recovery, compared this with how they had felt before stroke. Others had succeeded to negotiate a more positive identity which included their changed abilities. In a previous study [32] it was concluded that individuals require time to come to this realization. It has also been argued that social support in everyday situations may be important [35]. Supportive reactions from others mirror the person with stroke in their way of being or acting, and this might contribute to a positive and important reality adjustment for the individual [35].

In our study, close relationships were described as positive resources during the process of forming new daily routines and reaching greater acceptance of consequences after stroke. However, due to increased discomfort when socializing after stroke, some had resigned to a more passive social role or felt that friends had withdrawn. Participants who perceived lack of social support found it more difficult to cope with the recovery processes. Being socially isolated may generally enhance feelings of hopelessness and disconnection in life [29].

Most studies included in a review article on identity following stroke rehabilitation [32], illustrated the importance of social support. It was also shown in a qualitative article [36] that, five years after stroke, participants had far fewer friends than before which was referred to as a major loss. In our study, difficulties concentrating, or fatigue had led to frustration and a less active social life. This kind of daily adjustment, to avoid being worn out have been referred to as negotiations between the lived body, participation in everyday life and sense of self [37].

Participants asked for more rehabilitation but were uncertain about what kind of help they were entitled to, and which services they needed to self-fund. It has been argued previously [38] that in countries with universal health care, difficulties can arise when negotiating complicated systems, and that patients often self-fund as a result. In the present study, concerns about inadequate, inaccessible or poorly coordinated health care services were raised. Consistent with our findings, the vulnerability of individuals who experienced a lack of help from the health care system has been described in a case study [39]. In addition, it was found [40] that persons with stroke wanted to develop supportive relationships with health professionals on their way to recovery. A review [41] showed that relationships with health professionals should be collaborative, with the professional listening to the individuals’ needs, giving expert advice and answering questions. Since the current study found that participants many years after stroke still experienced improvements through persistent rehabilitation, and wished for a more interactive rehabilitation training, collaborative forms of rehabilitation are suggested in the long-term phase of stroke.

Our findings showed a great need for accessible counselling or psychology services, which was shown before [41]. Participants who needed cognitive tools and structure to cope in everyday life, did not get help because they were unable to attend appointments. Consequences like fatigue, headaches or problems with short-term memory made it complicated to attend, or even cancel appointments on time. Therefore, psychology services need to be tailored to suit the individuals they aim to help, and home visits might be worth considering.

The strengths of this study were that photovoice proved to be an effective method for actively engaging individuals in issues about their lives after stroke. Participants emphasized that the current photovoice study had provided an opportunity for them to reflect about what topics were most meaningful to convey about their lives after stroke. Participants stressed that the focus group discussions had been rewarding; it was interesting to discuss mutual concerns, to see others’ photos and to hear their stories. The set-up with group discussions also contributed further reflections, which participants appreciated.

In this study, the photovoice method was valuable in visualizing everyday situations in a clear and direct manner. Participants selected interesting motifs to photograph, which will probably be easily remembered by potential readers. Photographs contributed to rich and collaborative focus group discussions. It remains to be seen whether this method will improve dissemination of results outside the field of research, and whether results will be less likely to submerge in the mass of articles about life after stroke. The action research design allowed for participants to have a voice in how to present their results (photos) in discussions as well as in the exhibition [15].

The photovoice method is demanding for participants, which can be seen as a limitation. Participants invest a lot more time and engagement, compared to what is required in a more traditional qualitative interview study [13]. In the current study, participants took part in a meeting about the study, which included ethics and camera training. They participated in a focus group and gave feedback on results, and on photovoice as a method. In addition, they were encouraged to reflect upon their lives so that they could select appropriate photography motifs, and finally; they prepared themselves for what to share in the focus group. As these efforts were not met with any financial compensation, it is important that participants feel motivated, and preferably that they see their participation as meaningful for the study. Participants in this study expressed a personal interest in questions relating to circumstances after stroke, on a personal as well as a societal level. A possible limitation with this study is that the study group might have a higher general interest in concerns regarding disability policies and science, in comparison to other persons with stroke. It is however difficult to speculate on in what ways this might have affected the results.

## Conclusions

The contribution of this photovoice study was illustrations of an altered way of living in a long-term perspective after stroke. Most participants had adapted or changed approaches in everyday situations and had thereby regained a sense of control in life. However, it was clear that mental processes towards acceptance in life after stroke were hindered by depression and that some individuals felt alone in this ongoing struggle. This shed some light on depression, social isolation and a resigned state of mind that might be neglected or not followed up sufficiently in long-term stroke surveillance. We urge future research to investigate the role of specialists in facilitating long-term adaptation processes after stroke.

Furthermore, long-term healthcare services and other interventions aimed to help persons with stroke should place more emphasis on addressing their needs. It was clear that available interventions a long time after stroke were not flexible enough to meet all individuals’ needs.

## Supporting information

S1 Example images Participants’ photos with descriptions. (DOCX)
