# Supplementary material for: “My life after stroke through a camera lens”- A photovoice study on participation in Sweden
Source: PLoS One. 2019 Sep 11;14(9):e0222099. doi: 10.1371/journal.pone.0222099 (PMC6738637; doi:10.1371/journal.pone.0222099)
Supplement: S1 Example images — (DOCX) [file pone.0222099.s001.docx]

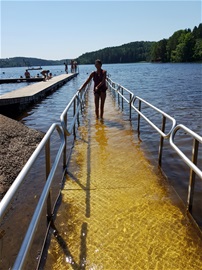

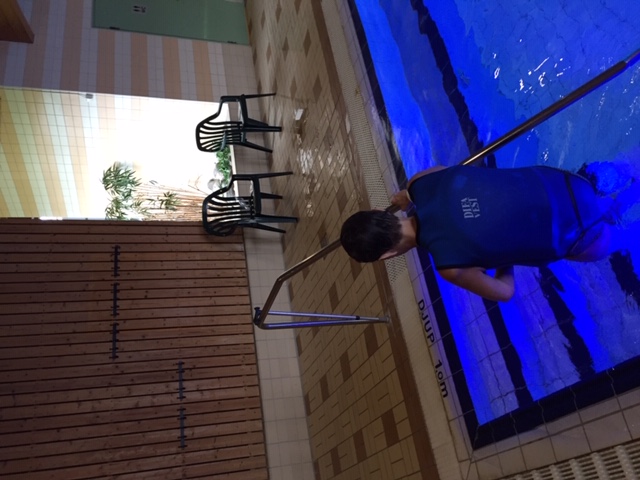


I love the sea and swimming, and working out in the pool


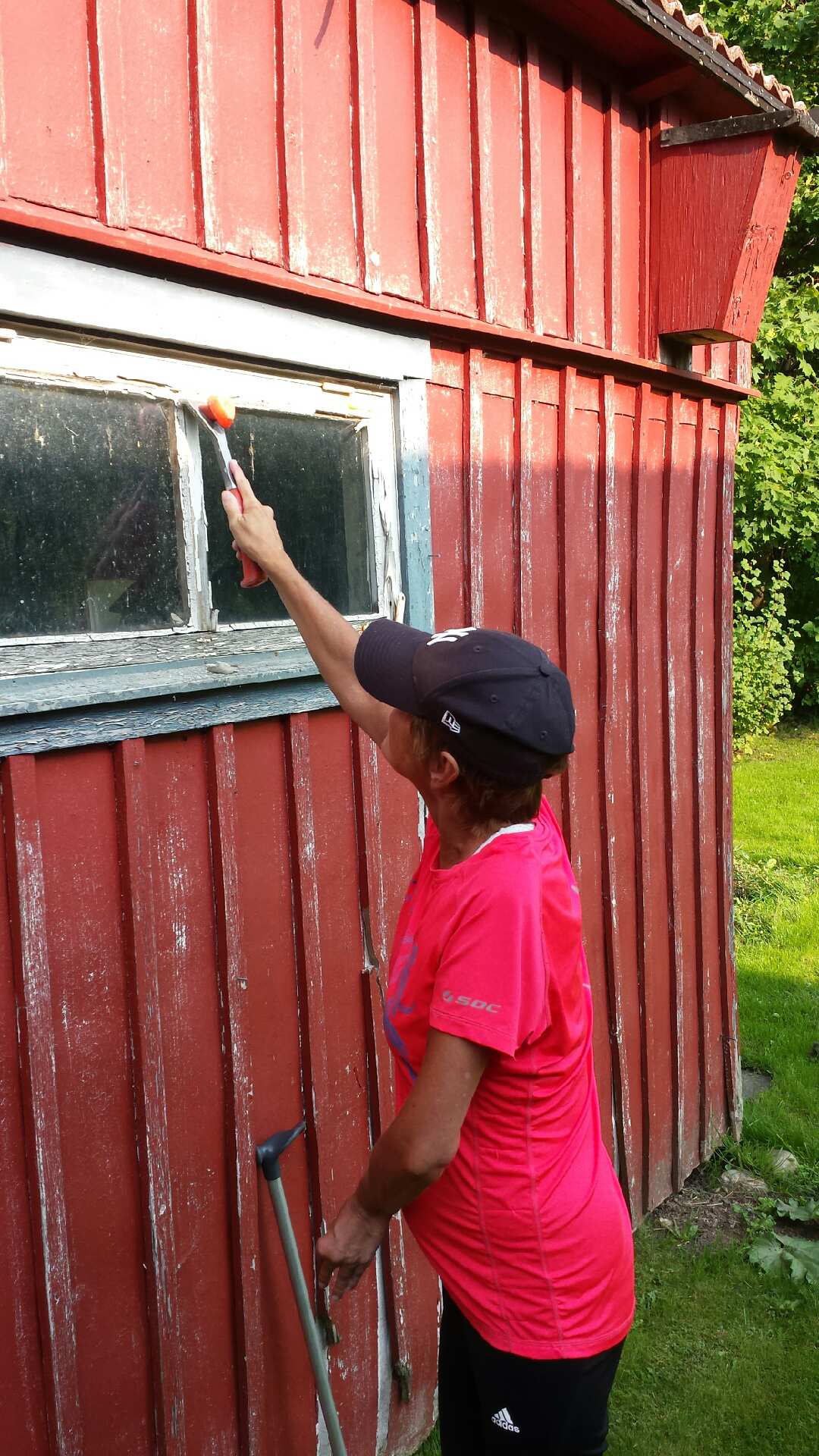

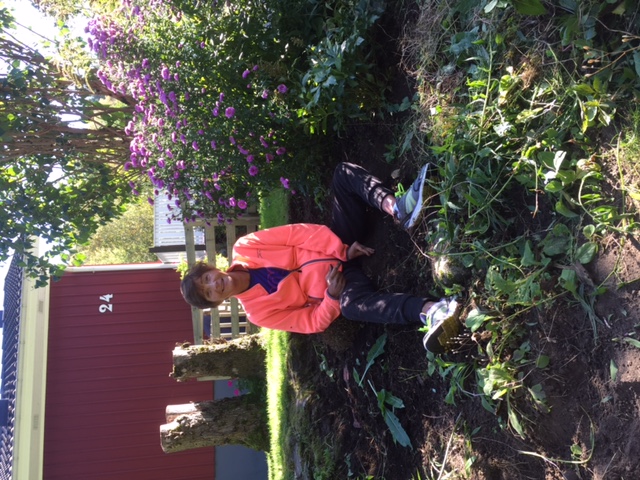


I like working in my country house


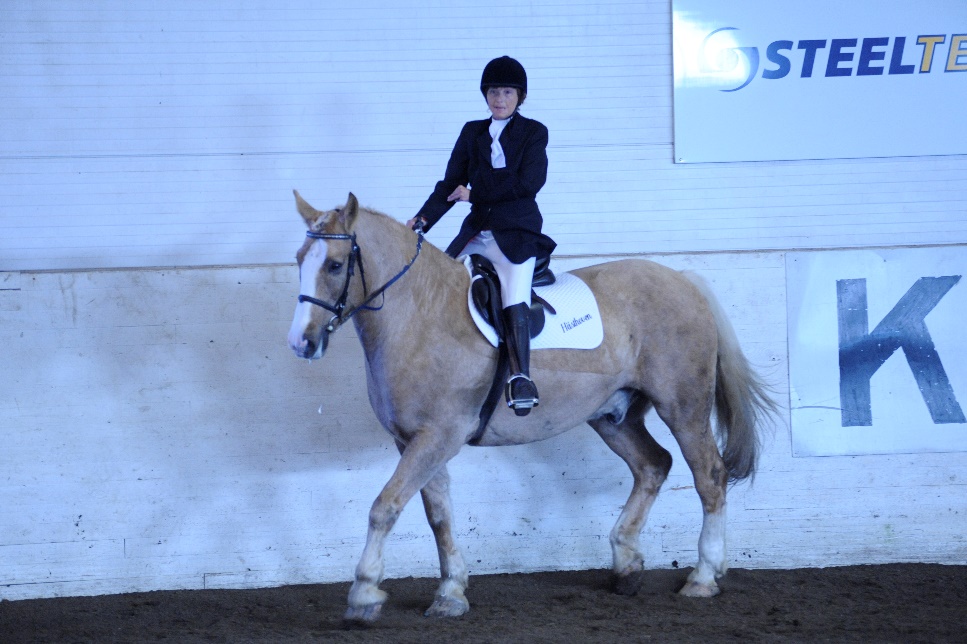


Riding a horse after stroke is no problem!


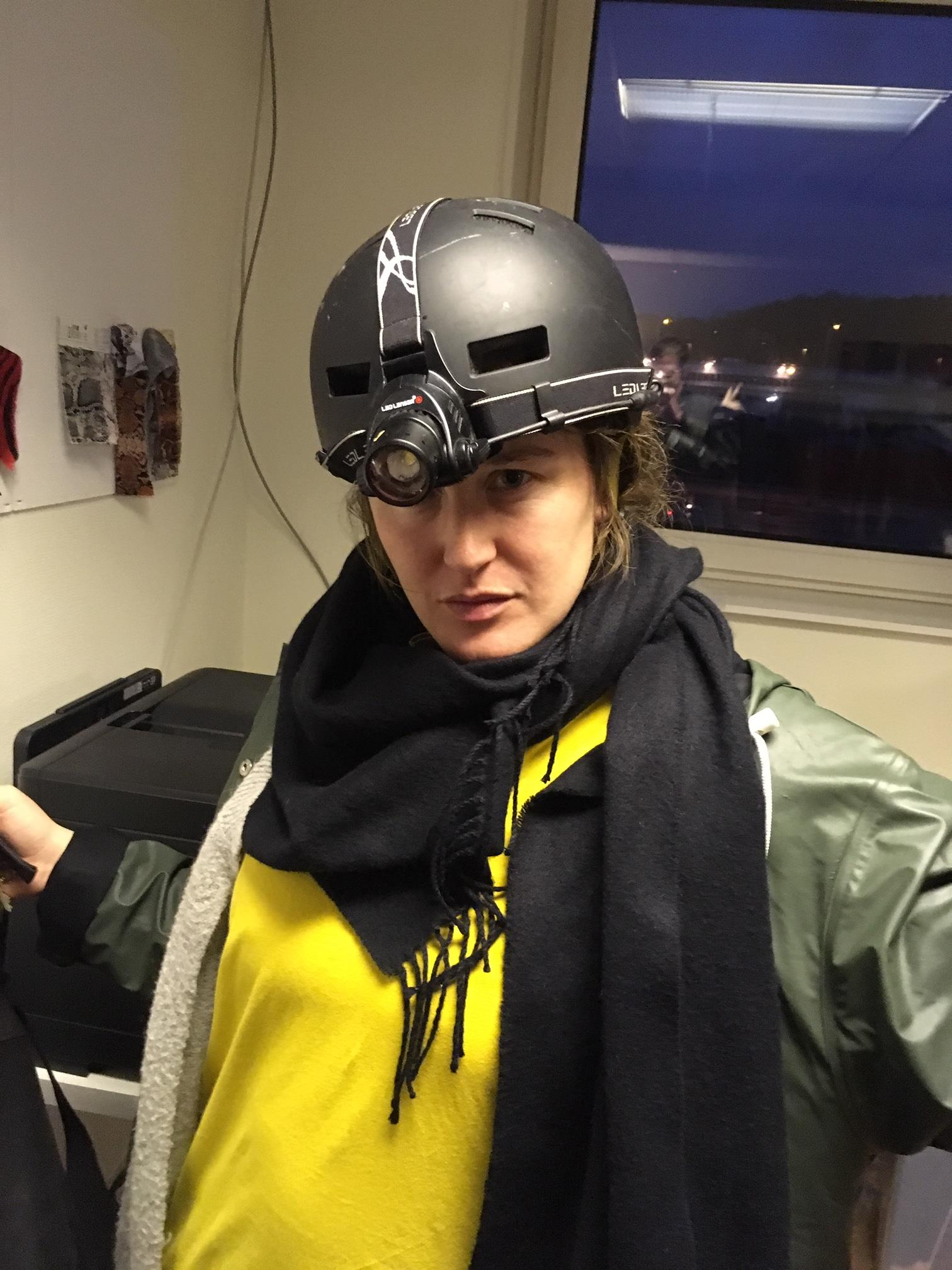


I can still ride my bicycle, even though my balance is not what it used to be


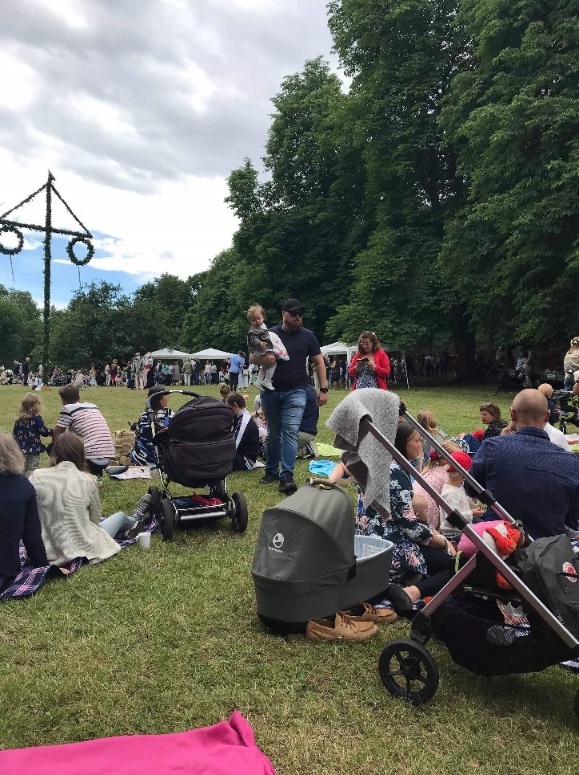

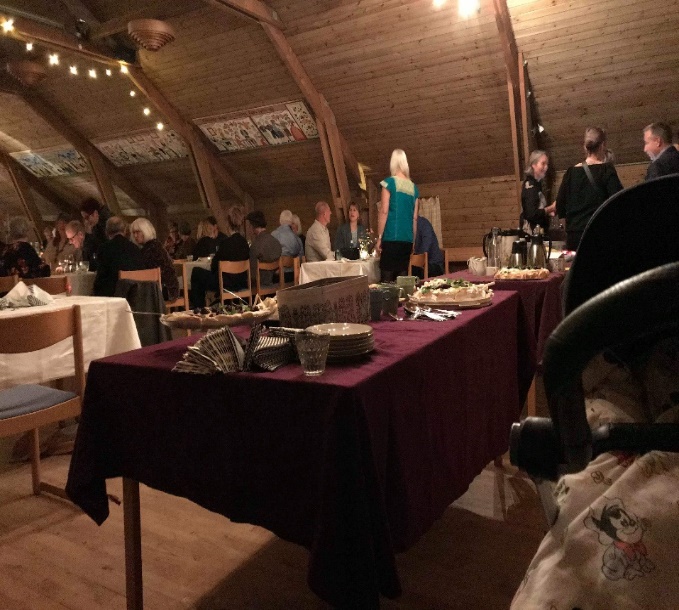


Social gatherings are not much fun anymore


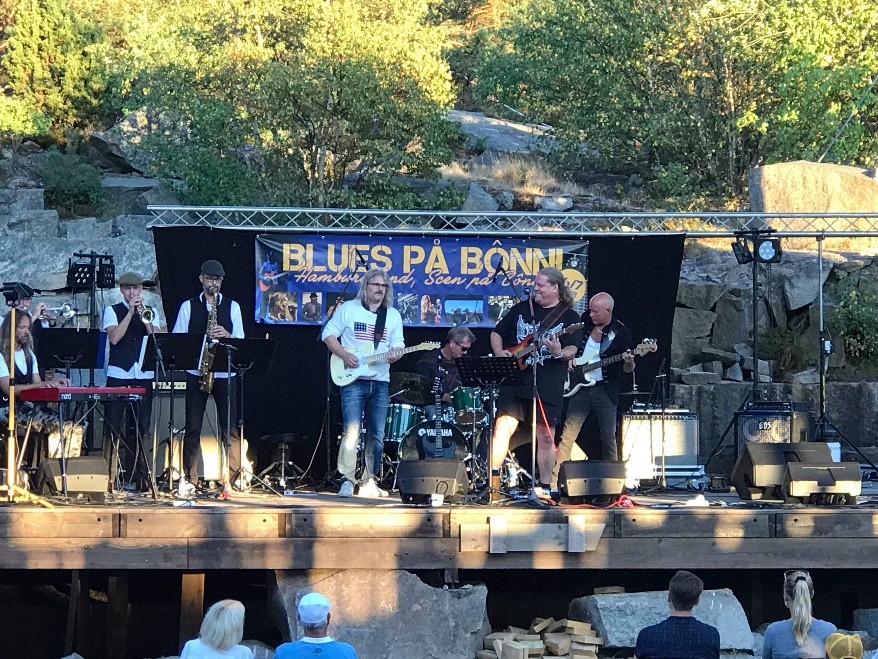


Music-concerts are more tiring now


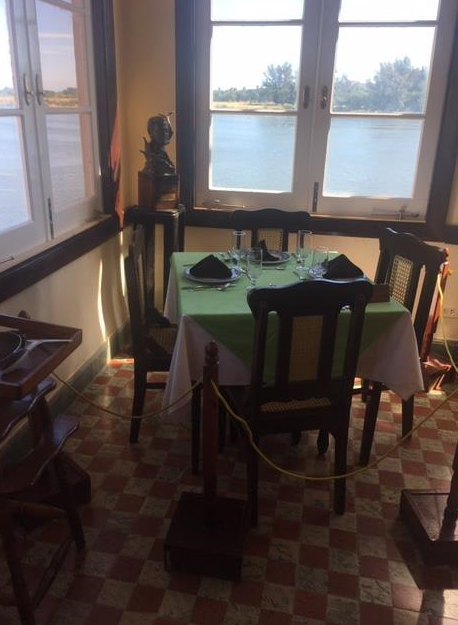


I miss going to a restaurant with my friends


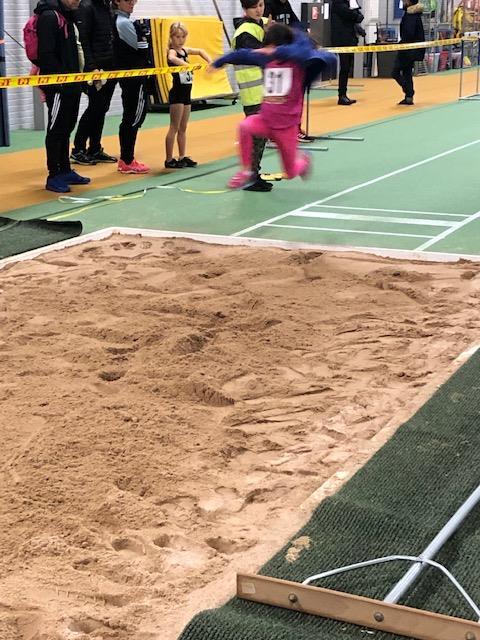

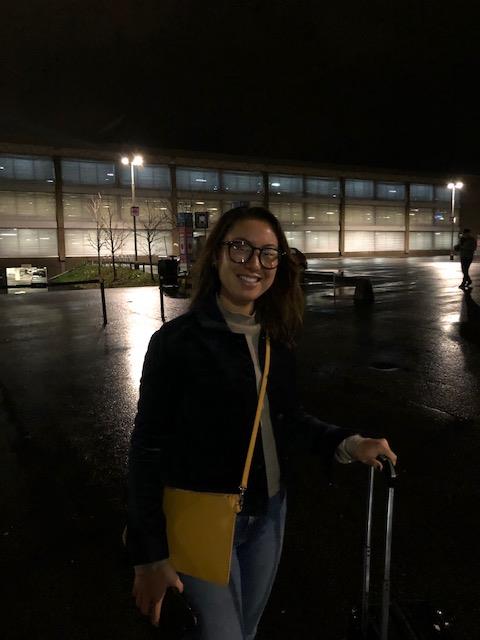


My daughter means the world to me


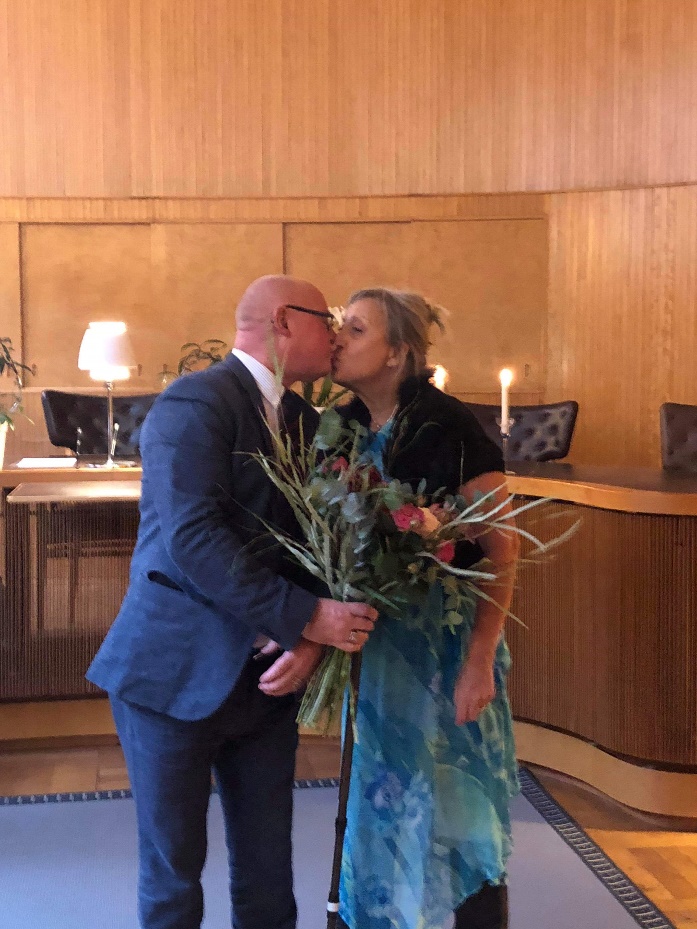


After the stroke, I got married!


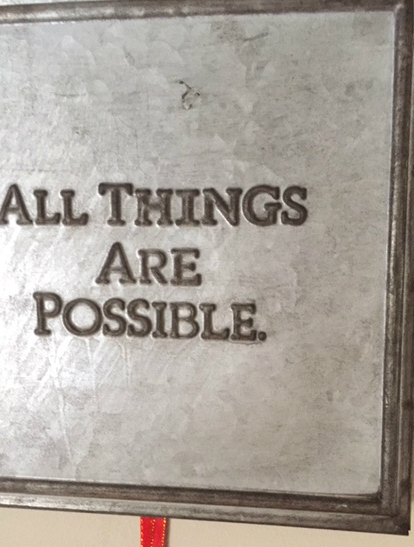


This is what I remind myself


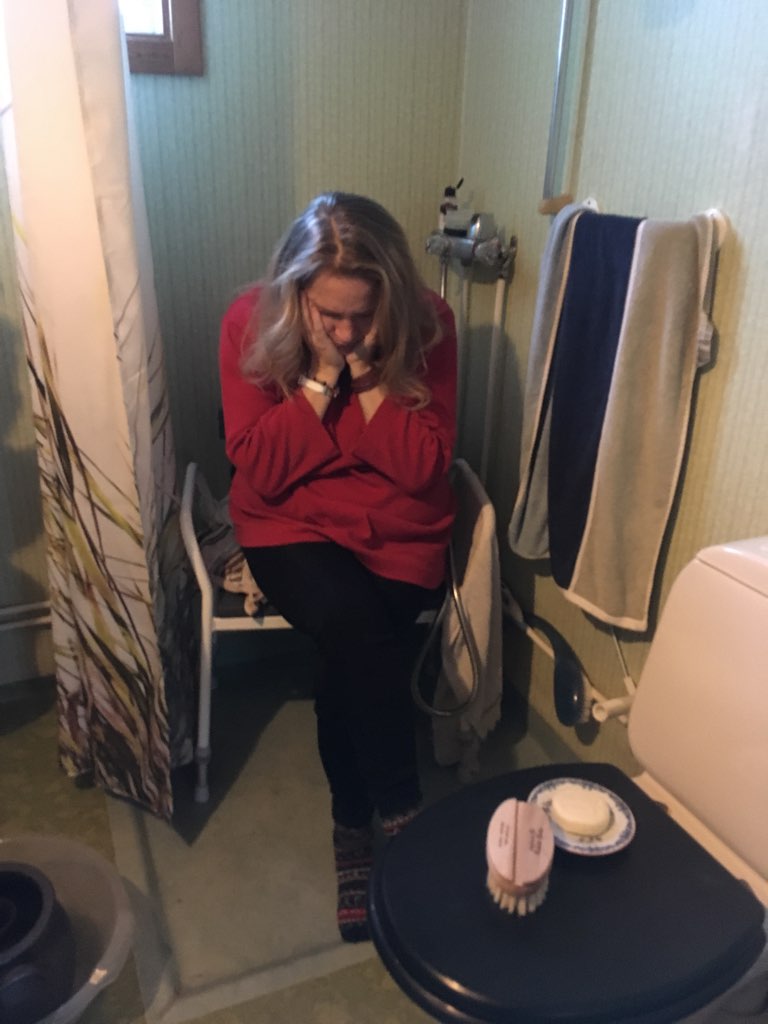


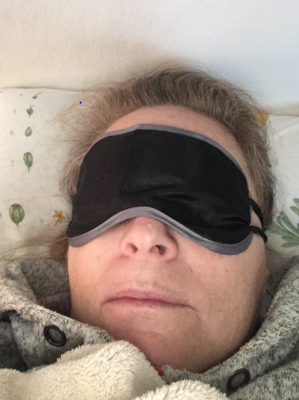


The headache is so painful, and I cannot live a normal life


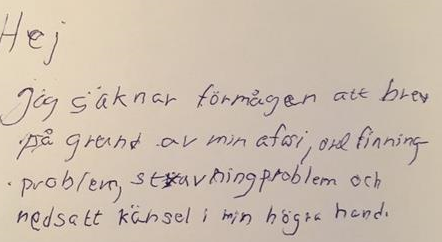


I miss my ability to write. Handwriting takes for ever.

My motorized wheelchair is my very own Statue of Liberty


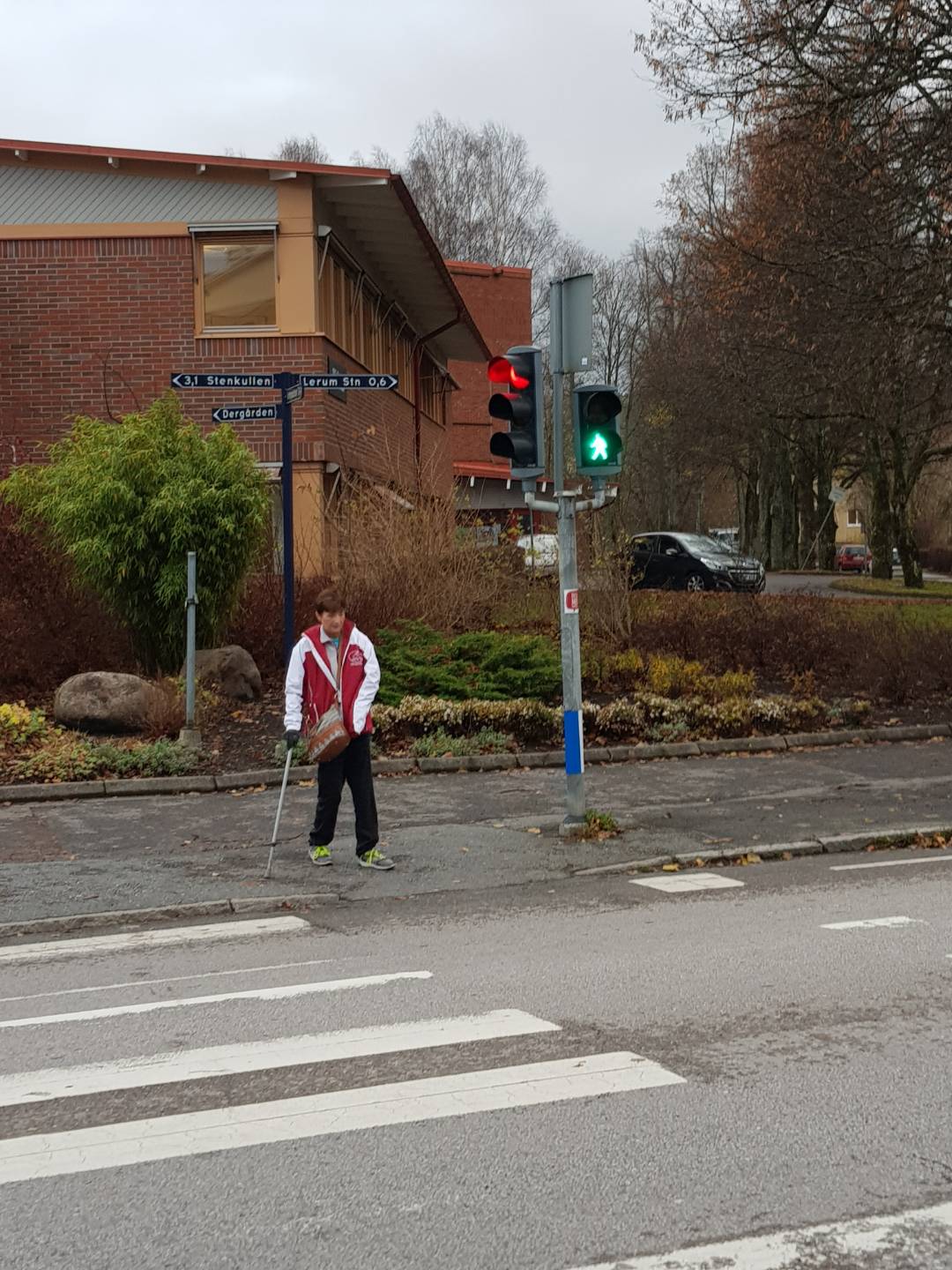

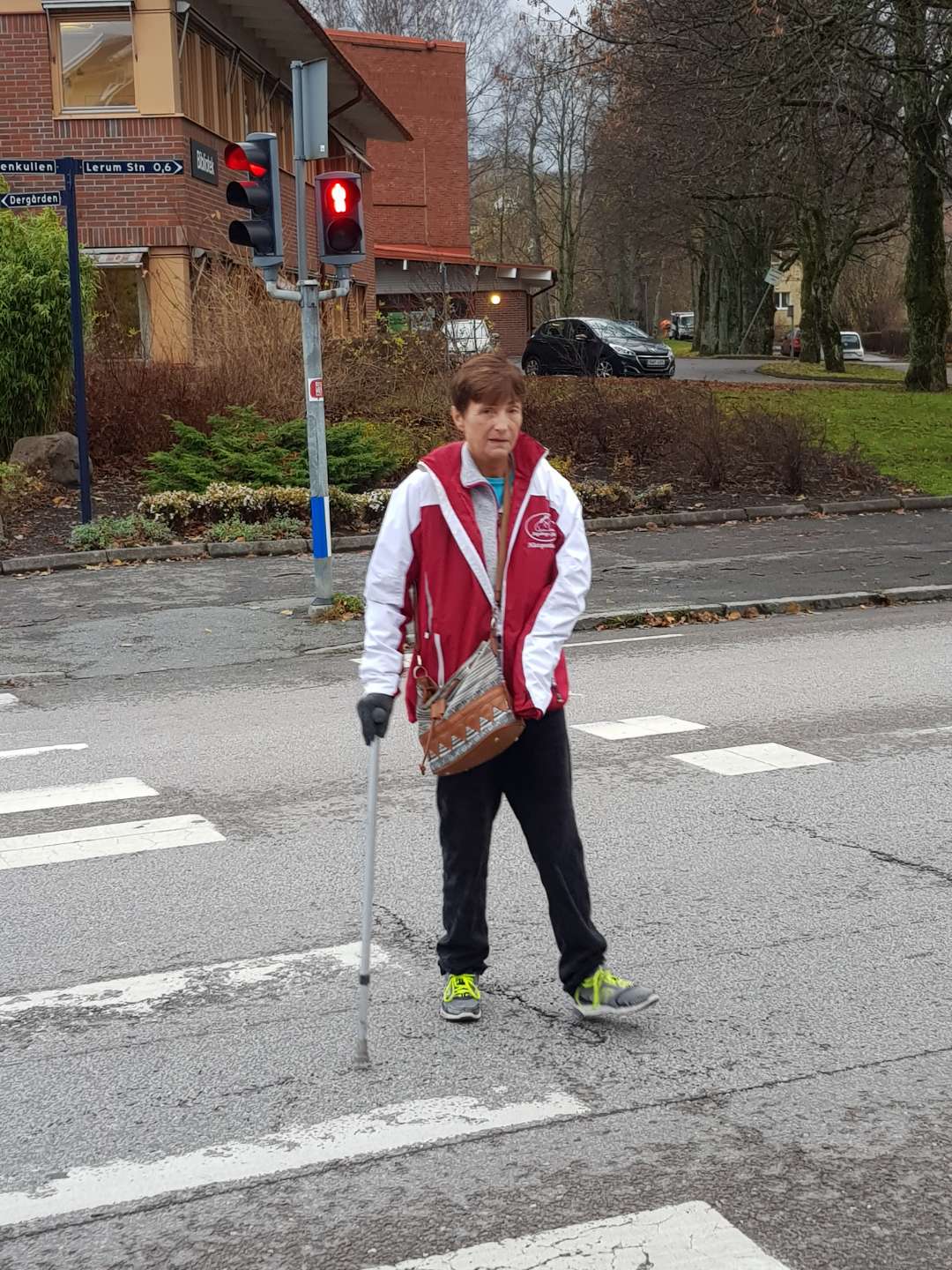


It is difficult to cross the road before the light switches to red


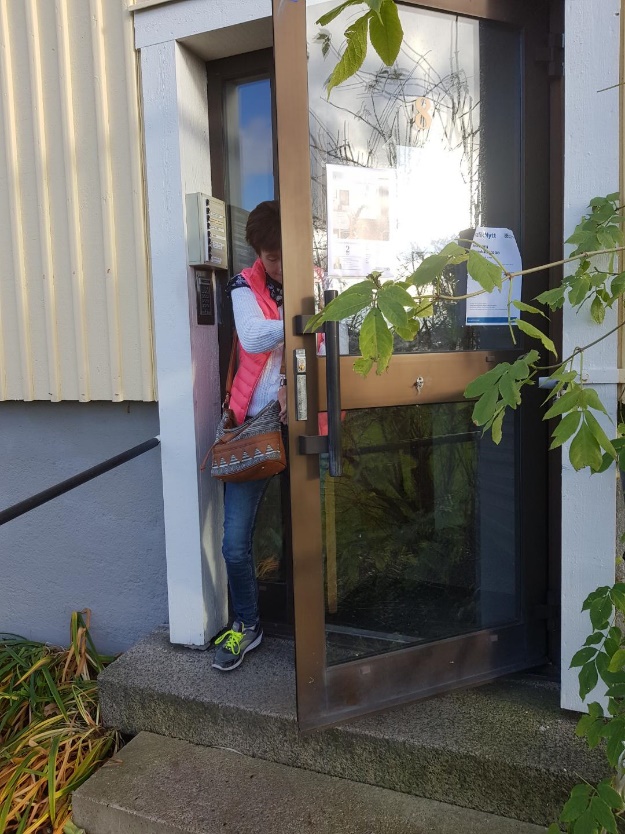


Heavy doors are obstacles


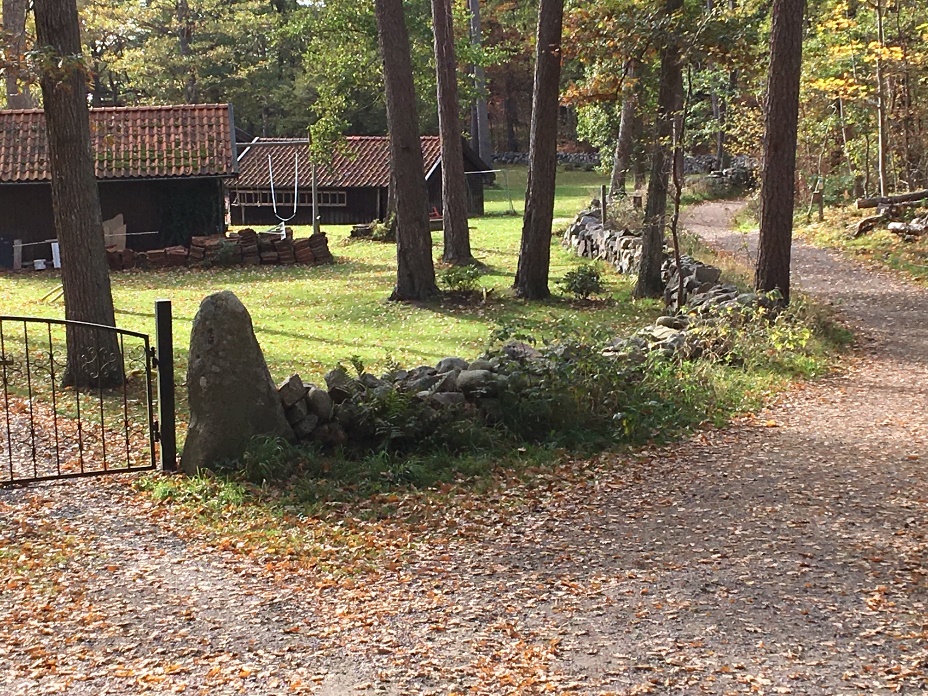


I like to walk in the nature, as long as the ground is even


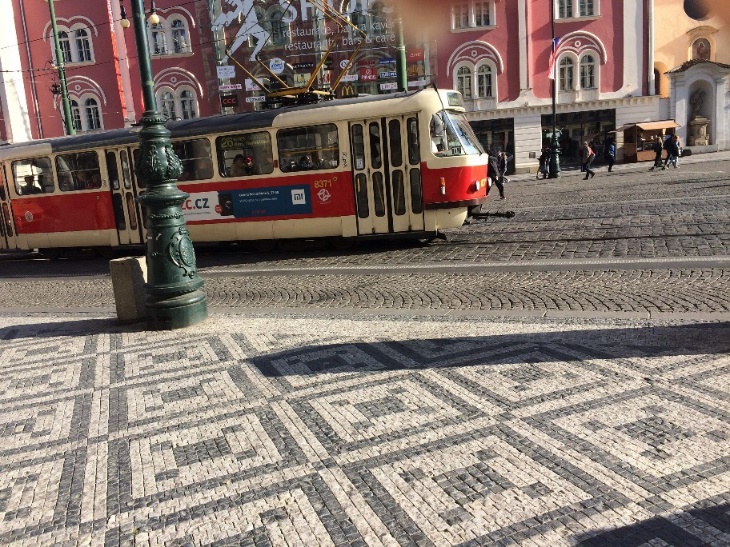

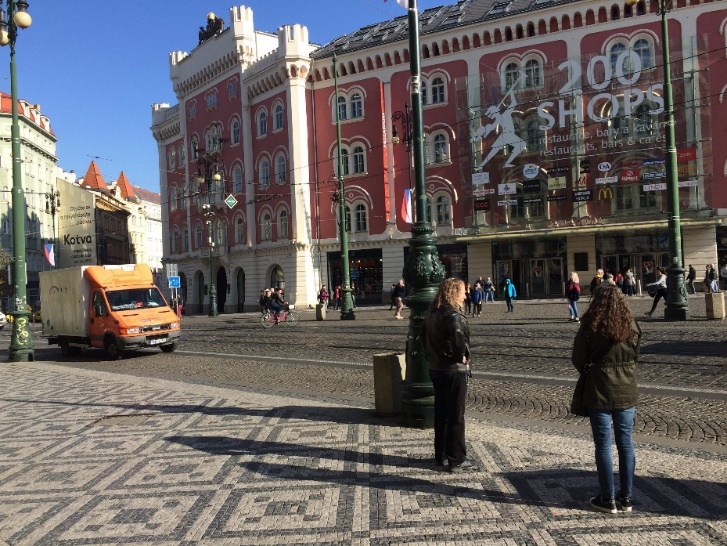


I want to be able to walk on a flat and smooth surface


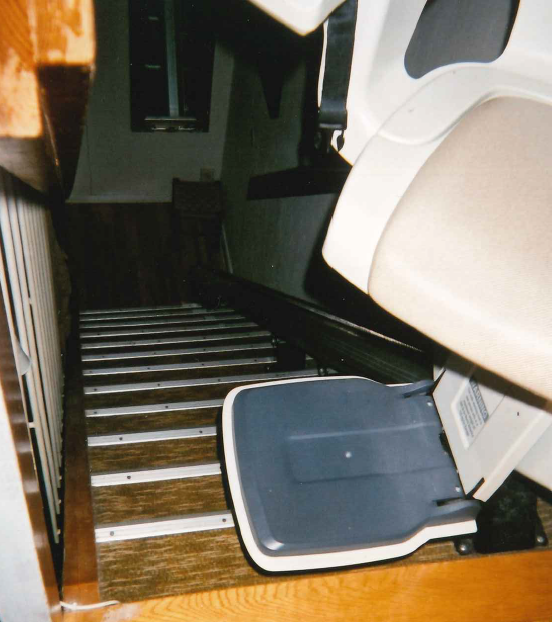


The steps are too high – that’s why I use a stair lift


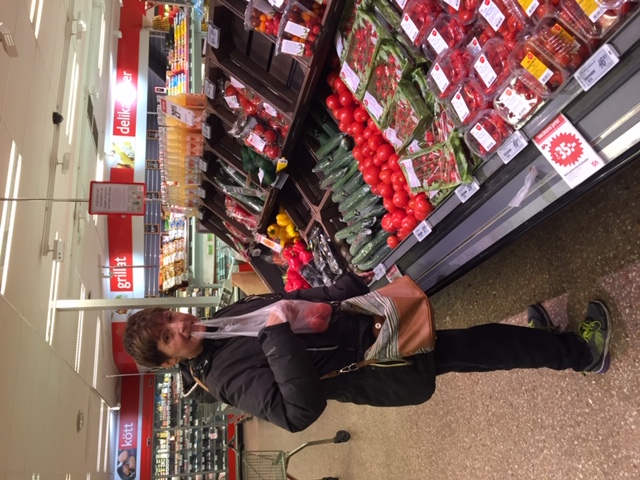


You need to be inventive when shopping for groceries


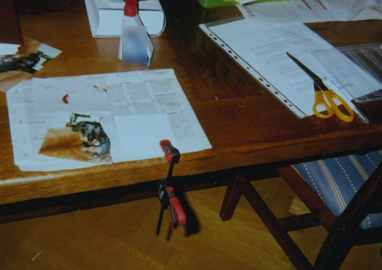


Using a one-hand operated clamp I can paste in photos in my album


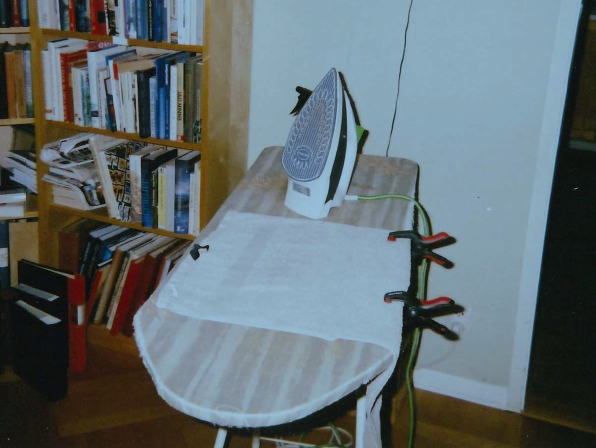


With one-hand operated clamps I can iron without any problems

Being able to cook again is really nice


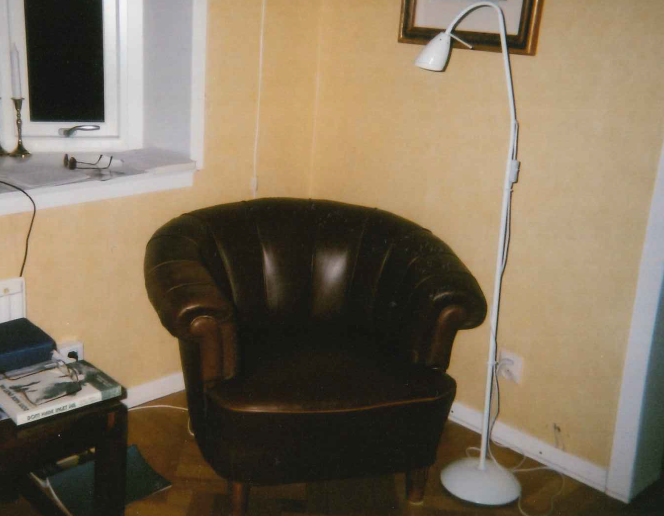


I love to sit in my chair and read, it is a quiet recreation
